# Analytical “bake-off” of whole genome sequencing quality for the Genome Russia project using a small cohort for autoimmune hepatitis

**DOI:** 10.1371/journal.pone.0200423

**Published:** 2018-07-11

**Authors:** Daria V. Zhernakova, Sergei Kliver, Nikolay Cherkasov, Gaik Tamazian, Mikhail Rotkevich, Ksenia Krasheninnikova, Igor Evsyukov, Sviatoslav Sidorov, Pavel Dobrynin, Andrey A. Yurchenko, Valentin Shimansky, Irina V. Shcherbakova, Andrey S. Glotov, David L. Valle, Minzhong Tang, Emilia Shin, Kathleen B. Schwarz, Stephen J. O'Brien

**Affiliations:** 1 Theodosius Dobzhansky Center for Genome Bioinformatics, St. Petersburg State University, St. Petersburg, Russian Federation; 2 University of Groningen, University Medical Center Groningen, Department of Genetics, Groningen, the Netherlands; 3 Research Resource Center for Molecular and Cell Technologies, Research Park, Saint-Petersburg State University, St. Petersburg, Russia; 4 Institute of Genetic Medicine, Johns Hopkins University School of Medicine, Baltimore, MD, United States of America; 5 Wuzhou Red Cross Hospital, Guangxi, China; 6 Pediatric Liver Center, Department of Pediatrics, Johns Hopkins University School of Medicine, Baltimore, United States of America; 7 Guy Harvey Oceanographic Center, Halmos College of Natural Sciences and Oceanography, Nova Southeastern University, Fort Lauderdale, Florida, United States of America; German Cancer Research Center (DKFZ), GERMANY

## Abstract

A comparative analysis of whole genome sequencing (WGS) and genotype calling was initiated for ten human genome samples sequenced by St. Petersburg State University Peterhof Sequencing Center and by three commercial sequencing centers outside of Russia. The sequence quality, efficiency of DNA variant and genotype calling were compared with each other and with DNA microarrays for each of ten study subjects. We assessed calling of SNPs, indels, copy number variation, and the speed of WGS throughput promised. Twenty separate QC analyses showed high similarities among the sequence quality and called genotypes. The ten genomes tested by the centers included eight American patients afflicted with autoimmune hepatitis (AIH), plus one case’s unaffected parents, in a prelude to discovering genetic influences in this rare disease of unknown etiology. The detailed internal replication and parallel analyses allowed the observation of two of eight AIH cases carrying a rare allele genotype for a previously described AIH-associated gene (*FTCD*), plus multiple occurrences of known *HLA-DRB1* alleles associated with AIH *(HLA-DRB1-03*:*01*:*01*, *13*:*01*:*01 and 7*:*01*:*01*). We also list putative SNVs in other genes as suggestive in AIH influence.

## Introduction

In the last decades whole genome sequencing (WGS) has become widely used in genomic studies. WGS technology improvement and decreasing sequencing costs have led to its increasing usage in medical diagnostics. As a consequence more and more groups set up new sequencing facilities to enable processing their samples in-house. A commonly used alternative is to outsource the sequencing, sending samples to a well-established sequencing center. Comprehensive comparisons of different next-generation sequencing (NGS) technologies have been performed to-date [1–5], aiding to the choice of NGS platform depending on the purpose of the study.

The Genome Russia Project will gather blood samples of some 3500 Russian people, including several hundred family trios (DNA samples of a child and both parents). The project will create a national collection of genetic data and engage researchers from multiple educational institutions and research organizations [6,7]. Genome Russia will reach across Russian Biomedical Centers and join with the international “1000 genomes project” created to uncover rare gene variants in different human populations [8]. DNA from the Russian volunteers will be subject to whole genome sequencing (WGS) suitable for estimating population-specific allele frequencies of determinants of complex chronic and infectious diseases with a genetic underpinning.

Genome Russia is important not only for the medical field and healthcare but also for biologists, political scientists, ethnographers, and historians, since we shall compile a comprehensive DNA variant information database for major ethnic groups living on the Russian territory. Population genetic analyses will enable historians and ethnographers to achieve better understanding of historic movements of ethnic groups, while pharmacists and clinicians will access data on efficacy of different medical drugs for different people, a beginning to precision medicine in Russia.

Recently, a new sequencing center has been set up at Peterhof (St. Petersburg State University, Russia) to provide sequencing facilities for various research projects including Genome Russia. Here we aimed at evaluating the performance of this center to determine whether it is suitable for sequencing thousands of human genomes within Genome Russia Project in comparison with commercial sequencing centers abroad.

We evaluated the WGS quality, efficiency and reproducibility of sequences obtained from the newly established core sequencing facility at Peterhof and from two established sequencing centers: Illumina (UK) and Macrogen (Seoul, Korea). Each center received DNA from the same ten individuals (8 clinical cases and two healthy parents of one case) who were involved in a disease association study targeting autoimmune hepatitis (AIH). Each facility provided 30x coverage of the same ten individuals with no financial charge, as they wanted to be considered for sequencing the volunteers collected by the Genome Russia Consortium.

We report here the detailed quality control analyses performed for each center, the efficiency of SNV and genotype calling, genotype comparison with each other and with DNA array chips of the same patients, the assessment of copy number variation and the speed of WGS thoughput promised. These data are offered with explicit recommendations for the Genome Russia sequencing based upon our interpretation. Additionally, we examined variants predicted as loss-of-function within the AIH cases in a prelude to discovering genetic influences in this rare disease of unknown etiology.

Our analysis highlights the differences in various aspects of resulting data between commercially available sequencing providers and thus will be of use not only for the newly established sequencing centers, but also for those who outsource their DNA-sequencing.

## Results

### I. Comparison of whole genome sequencing results

#### Sample description and data generation

Sequencing was performed on a newly generated dataset of ten samples, of which eight (3 males and 5 females) were patients having autoimmune hepatitis and two were non-affected parents of one of the patients (Table 1).

**Table 1:** Sample description.

Sample	Diagnosis	Gender	Ethnicity*	Age at biopsy/diagnosis	Sequenced by**		
					M	I	P
trio_mother	Healthy	F	EA	NA	+	+	+
trio_father	Healthy	M	EA	NA	+	+	+
trio_case1	AIH-type II	F	EA	19 months	+	+	+
case2	AIH-type I	F	EA	6 years	+	+	-
case3	AIH-type I	F	EA	20 months	+	+	-
case4	AIH-type I	F	IA	11 years	+	+	+
case5	AIH-type I	F	EA	15 years	+	+	+
case6	AIH-type I	M	AA	8 years	+	+	+
case7	AIH-type I	M	EA	17 years	+	+	-
case8	AIH-type I	M	EA	12 years	+	+	-

Phenotype information for 10 samples under study. Last four columns show whether a sample was sequenced at the corresponding sequencing center (+) or not (-).

*EA—European American; IA—Native American; AA—African American

Sequencing centers:

**M—Macrogen-X10; I—Illumina-X10; P—Peterhof-HiSeq4000

In order to compare the results of sequencing performed by different sequence providers, the same six samples were sequenced at three sequencing centers Illumina-X10 (UK), Macrogen-X10 (Korea) and Peterhof-HiSeq4000 (Russia). Illumina-X10 and Macrogen-X10 sequenced all 10 samples (see Table 1).

#### Quality control of raw reads

**Fig 1:**
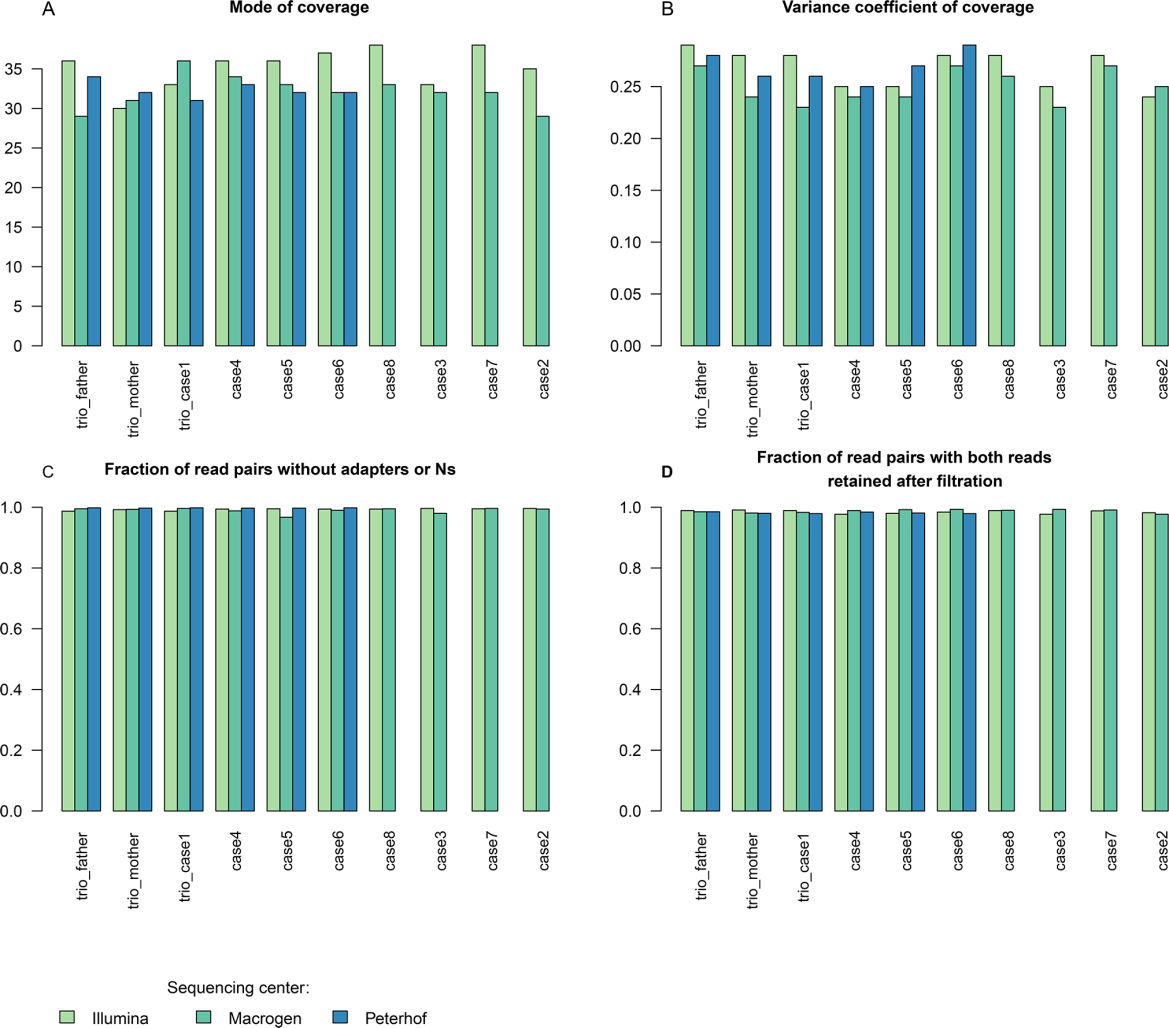
Raw read quality control parameters. Raw sequence read QC parameters are shown for three sequencing centers (colored differently).

Comparison of raw data quality must take into account the presence of adapters, read quality, error rate, coverage of target genome and uniformity of coverage. To that aim we have chosen five parameters to compare: fraction of read pairs without adapters or Ns, fraction of read pairs with both reads retained after filtration, fraction of 23-mers with errors, estimated mean coverage and variance coefficient of coverage (see Methods). These parameters were used to set up criteria for including sequenced samples (see Methods for the detailed description). Histograms and values of corresponding parameters are presented in Fig 1, Table 2 and S1 Table.

**Table 2:** Comparison of sequencing results (N = 17 parameters).

	Parameter	Macrogen-X10	Illumina-X10	Peterhof-HiSeq4000
Sequencing strategy	Library preparation kit	Illumina TruSeq DNA PCR-Free	Illumina TruSeq DNA PCR-Free	Illumina TruSeq DNA PCR-Free
	Insert size	300–400 bp	450 bp	400 bp
	Read length	151bp, paired-end	151bp, paired-end	150bp, paired-end
Raw read QC	Estimated mean coverage	31.685	36	32
	Variance coefficient of coverage	0.245	0.28	0.27
	Fraction of read pairs with both reads retained after filtration	0.989	0.986	0.981
	Fraction of kmers with errors	0.076	0.068	0.069
	Fraction of read pairs without adapters or Ns	0.994	0.994	0.998
Mapping QC	Reads before mapping	812,203,657	834,018,799	912,695,503
	Percentage of mapped reads	97.85%	97.14%	97.43%
Variant QC	Number of SNVs	3956042	3971375	3552604
	% of novel SNVs	2.01%	2.05%	1.64%
	Number of indels	459983	708225	335164
	# Multiallelic sites	30180	122066	14031
	Mendel errors	0.58%	0.30%	0.27%
	Genotype concordance with microarray	96.80%	96.88%	96.67%

Main parameters used for comparison of sequencing centers are presented in this table. These and additional parameters can also be found in S1–S3 Tables. All sequenced samples were used in this comparison.

Read coverage is on average the highest in Illumina-X10, followed by Macrogen-X10 and Peterhof-HiSeq4000 (Fig 1A, Table 2, S1 Table). The significance of this difference is illustrated by the mean coverage by both ANOVA (p-value = 0.0041) and Kruskall-Wallis test (p-value = 0.009609) (Fig 1A). Pairwise tests also show this trend (Illumina-X10 vs Macrogen-X10 p-value = 0.00031, 95% CI [1.51, 4.92]); Illumina-X10 vs Peterhof-HiSeq4000 data (p-value = 0.00101, 95% CI [1.41, 5.35]). As the lower boundary of confidence interval is less than 1.6 (i. e. only approximately 5% of the target 30x coverage) this measure is not important for our purposes.

Statistically significant differences between facilities were also detected for variance coefficient of coverage (Fig 1B) by Kruskal-Wallis test (p-value = 0.04262; ANOVA is inapplicable for this parameter). Pairwise comparison of variance coefficient of coverage showed a significant difference only between Illumina-X10 and Macrogen-X10 (p-value = 0.02105). Macrogen-X10 had lower values of variance coefficient (which is calculated as a ratio of standard deviation to mean value), which means more uniform coverage, while Peterhof-HiSeq4000 data fell between Macrogen-X10 and Illumina-X10 datasets (Fig 1B, Table 2, S1 Table).

We detected no statistically significant difference between the datasets for the fraction of read pairs with both reads retained after filtration and the fraction of 23-mers with errors (Table 2, S1 Table) as tested under a parametric model (ANOVA mixed model p-values 0.0930, 0.3291) or a nonparametric model (Kruskal-Wallis test p-values 0.075, 0.4209).

Statistically significant difference for fraction of read pairs without adapters or Ns (Fig 1C, Table 2, S1 Table) was detected only by Kruskal-Wallis test (p-value = 0.0011) and was not supported by ANOVA (p-value = 0.0697). This discrepancy was possibly due to fewer samples (N = 6) sequenced by the Peterhof-HiSeq4000 facility, thus making the parametric test not able to detect the difference. Pairwise tests showed a small but significant difference in the comparisons of Illumina-X10 vs Peterhof-HiSeq4000 (p-value = 0.001) and Macrogen-X10 vs Peterhof-HiSeq4000 (p-value = 0.001045).

#### Read alignment

In order to compare genomic variation, we first aligned reads to the human reference genome. Our dataset contained two samples of non-European descent (Native American and African American). All samples irrespective of ethnicity were aligned to the same human reference genome, due to the absence of an alternative reference genome, and presuming this is not likely to substantially bias genotyping in non-repetitive regions [8]. Indeed, the percentage of mapped reads was not substantially different in the non-European samples.

Overall, alignment rate was similar across the three sequencing centers yielding around 97.5% of reads mapped (Table 2). We first aligned all datasets using the same default parameters of bowtie2 aligner. This resulted in discordant read pairing in 31% of read pairs in Illumina-X10 dataset. After detailed investigation we found that this happened due to a larger insert size of Illumina-X10 reads (see Table 2) than the default insert size used by bowtie2. To overcome this we increased the bowtie2 insert size to 800bp, which indeed increased the percentage of properly paired reads to 95%. The results obtained for other datasets with the default settings were acceptable; therefore we did not rerun bowtie2 with an increased insert size for the other datasets. This resulted in slightly lower percentages of properly paired reads in Macrogen-X10 and Peterhof-HiSeq4000 as compared to Illumina-X10 (Table 2). Detailed mapping statistics for all datasets can be found in S2 Table.

#### Variant calling and genotyping

Alignments were then used for variant calling and genotyping. We performed joint genotyping on all sequenced samples separately in each dataset.

**Fig 2:**
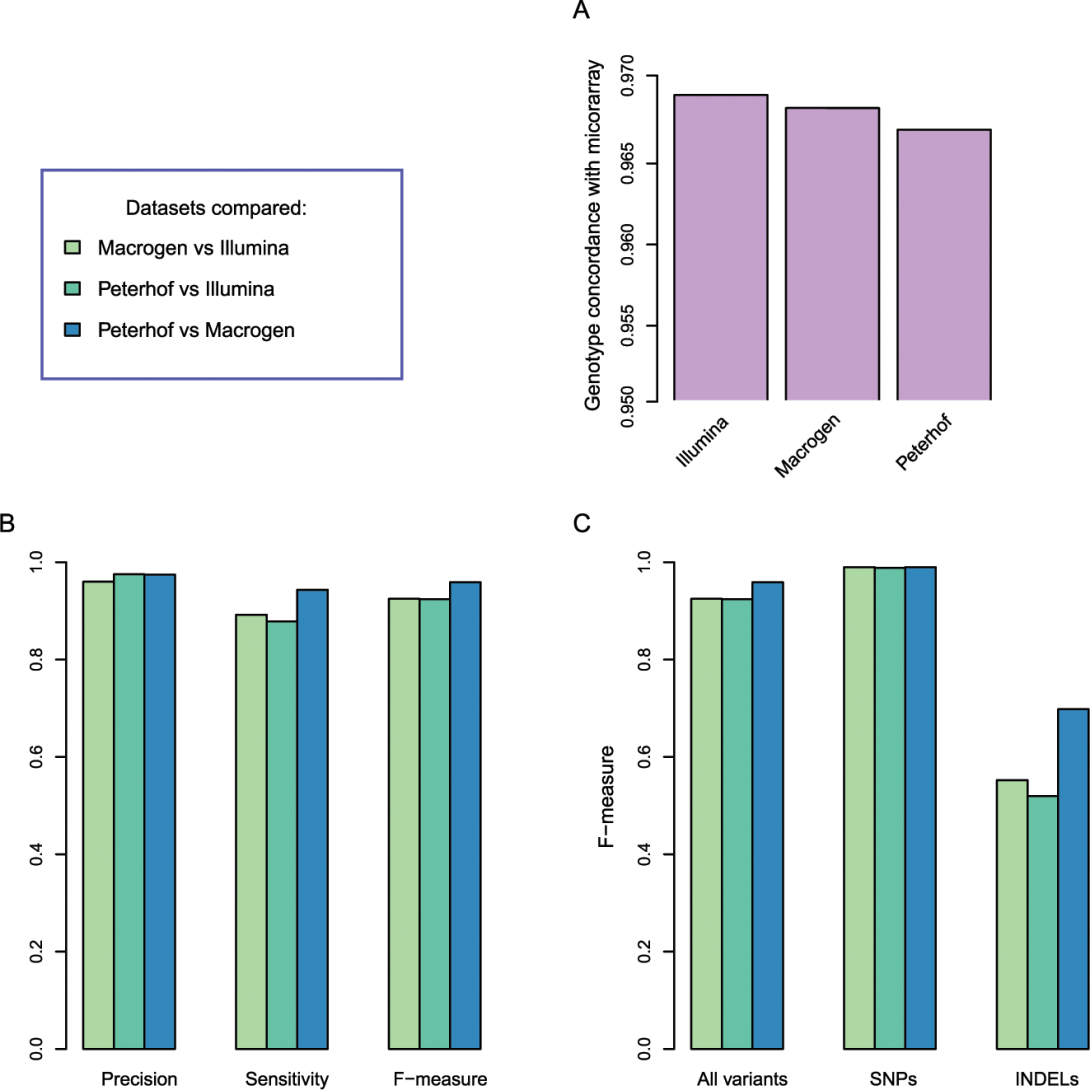
Genotype comparison. (A) Concordance of WGS genotypes with microarray genotypes. The concordance was estimated based on the trio data as the ratio of microarray SNPs with identical genotypes in WGS results. (B) Comparison of the three WGS datasets between each other in terms of precision, sensitivity and F-measure for pairwise comparisons. Color legend is given on the top right. (C) Concordance of genotypes in the three WGS datasets for all variants, SNPs and indels. Color legend is given on the top right.

Variant calling identified more than 3.5 million SNPs and more than 50,000 short indels (Table 2). Peterhof-HiSeq4000 data had the lowest number of identified variants partly due fewer samples (N = 6) compared to Illumina-X10 and Macrogen-X10 datasets (N = 10). This difference becomes lower when comparing only the 6 shared samples (S3 Table). We also compared the number of Mendelian inheritance errors based on trio genotypes and found a lower error rate in Peterhof-HiSeq4000 and inMacrogen-X10 (Table 2, S4 Table). Allele count distributions were similar across datasets (S1 Fig). To assess genotyping quality we used microarray genotypes of the trio provided by Illumina. It was previously shown that comparison of WGS with microarrays provides an accurate estimation of variant detection sensitivity [9]. We estimated the percentage of microarray SNPs that were correctly genotyped in sequencing datasets. All datasets detected more than 96% of microarray SNPs with the same genotypes, while Illumina-X10 showed the highest number of concordant SNPs (Fig 2A, Table 2). We also investigated per-sample genotype concordance rate to identify potential outliers, such as sample ethnicity (S5 Table). Sample ethnicity did not appreciably influence genotype concordance (S5 Table), likely reflecting that read alignment and subsequent genotyping of common variants are not dramatically sensitive to common population-specific genetic variation in line with previous studies [8]. As expected, the number of variants (and the percentage of singletons) is higher in African American sample, in line with our knowledge of human ancestry with maximal variation and private alleles found across African populations.

Variants genotyped in the three datasets show a high overlap, more than 86% of variants identified in one dataset could be found with the same genotype in another dataset (Fig 2B, S5 Table). Highest overlap was observed between Macrogen-X10 and Peterhof-HiSeq4000 variants. The overlap is higher for SNPs than for short indels (Fig 2C) probably partly due to alignment difficulties at indel borders, contributing to lower indel calling quality and increased discrepancies

#### Copy number variation and segmental duplication

The copy number values distribution is close to normal with expected value around 2, which corresponds to the diploidy of the genome (S2 Fig).

Segmental duplications (SD) were defined as regions larger than 1Kbp of increased average copy number value in comparison to the mean copy number value in control regions of the corresponding individual with correction for dispersion [10–12]. The results for the trio are shown in S3 Fig. We evaluated the number of segmental duplications present in patients but absent in the non-diseased individuals in each dataset. Overall, there are eight such shared duplications in Illumina-X10 dataset, two in Macrogen-X10 dataset, 40 in Peterhof-HiSeq4000 dataset. The fact that there were only 4 AIH patients sequenced in the Peterhof dataset compared to 8 AIH cases in the two other centers (Table 1) likely affected the quantities of common duplications. The segmental duplications identified in patients, but not present in the two healthy parents, do not overlap among different datasets.

### Long insertions and deletions

We also called long indels (20–100 bp) in the three datasets. Illumina-X10 and Macrogen-X10 yielded around 2,500 long indels, whereas 6 samples from Peterhof-HiSeq4000 had around 1,900 long indels (S6 Table). In each dataset, about 80% of long indels were previously reported variants, and about 20% were novel.

From Illumina-X10 and Macrogen-X10, we selected 6 samples corresponding to the 6 samples present in Peterhof-HiSeq4000 and compared the three sets of long indels called in these 6 samples. Illumina-X10, Macrogen-X10, and Peterhof-HiSeq4000 shared approximately 50% of long indels, whereas about 20% of long indels were unique in each set (S4 Fig). This variation in long indels may be explained by variation in read mapping affecting the calling process. Long indel call sets for the 6 samples shared three indels overlapping with exons, and all the three were previously reported indel variants located in *KTI12*, *BRCA1 and PKD1L2*.

### HLA genotyping

We investigated how well we can produce HLA genotypes based on sequencing results as compared to molecular HLA typing. We produced HLA genotypes using Athlates software for HLA-A, HLA-B, HLA-C and HLA-DRB1 genes (S7 Table). All three datasets showed a similar mismatch rate as compared with molecular typing results: Macrogen-X10 had the lowest number of mismatches (17) and Illumina-X10 had the highest number of mismatches (21). S7 Table

### II. Autoimmune hepatitis

The cohort used for sequencing results comparisons consisted of eight patients diagnosed with autoimmune hepatitis (Table 1). Autoimmune hepatitis (AIH) is a rare highly heterogeneous complex disease of the liver with unknown etiology [13–15]. AIH occurs both in children and in adults, more often affecting females than males. There are two distinct forms of AIH, type 1 and type 2, which differ by the presence of autoantibodies [16]. Genetic studies have identified several genetic variants increasing the risk of developing AIH. *HLA* class II *DRB1* alleles were found to be associated with AIH disease in various populations [17–20]. Variants in several genes outside of *HLA* were also associated with AIH susceptibility or progression: *CTLA4* [21,22], *FAS* [23], *VDR* [24], *TBX21* [25], *TNF2* [26], *SH2B3*, *CARD10* [27] and *FTCD* [28].

The number of patient samples (N = 8) in our data was too low for a robust gene association analyses, thus we annotated the identified SNPs and indels to produce a list of variants potentially having an impact on AIH disease. In this study we aimed to make use of multiple variant and genotyping replicates for each individual to filter errors and validate associated variants, thereby reducing false positive calls. We recovered 897 variants predicted to be high-confidence loss-of-function (LoF) by LOFTEE tool [29] (398 SNPs and 497 short indels; S8 and S9 Tables respectively). We further identified variants with reported AIH associations in GWAS catalog [30] and in HGMD [31], performed pathogenicity annotation by Gavin [32], and investigated the reported gene expression in liver according to GTEx [33]. S8 and S9 Tables provide an unabridged detailed description of these annotated SNP and indel variants respectively.

In a filtered gene list (S10 and S11 Tables) we selected rare variant alleles that occurred at least twice in the eight AIH patients, and excluded those with MAF > 0.01 in 1000G [8],ExAC [34], or gnomAD [34] databases. We retained SNPs with zero occurrence of alternative allele in the healthy parents (or up to two alleles when their offspring carried the variant). In order to provide an additional filtering criterion in variant prioritization, we also processed the same Illumina dataset using BWA-GATK pipeline to capture pipeline-specific mapping and genotyping errors.

We included only SNPs with genotypes that were > 98% concordant in Macrogen, Illumina, Peterhof, and Illumina GATK replication results, and also included those with rare homozygous cases. In S10 and S11 Tables we list relevant information for each LoF variant: 1) Chromosome; 2) Coordinates; 3) rs id number; 4) Reference and alternative allele sequence; 5) Number of gene isoforms (transcripts) this variant falls into; 6) Most severe genetic impact of the variant (e.g. gained stop codon, frameshift, splice effect etc.); 7) Novelty of the variant; 8) Mendel transmission error in trio; 9) Liver expression according to GTEx [25]; 10) Gene associations from GWAS catalog [30] and in HGMD [31] and 11). Gavin [32] pathogenicity prediction for each variant. After variant filtering according to these aspects (see Methods), we derived a short list of 54 SNPs and 27 indels, which offer potential for replication in a larger study (S10 and S11 Tables).

We compared these SNP and indel variants (S10 and S11 Tables) to genes identified in additional AIH patient exploration studies described in S12 Table. Briefly, we examined gene candidates derived from three separate AIH gene lists produced by studies on AIH onset: 1) 39 genes with segmental duplications among the 8 cases studied here; 2) 21 genes identified in two AIH patient trios with elevated incidence of homozygotes for rare alleles; and 3) genes implicated in AIH by previous studies (HLA-DRB1[17–20], *CTLA4* [21,22], *FAS* [23], *VDR* [24], *TBX21* [25], *TNF2* [26], *SH2B3*, *CARD10* [27] and *FTCD* [28]).

We observed among the AIH cases two genes that were reported previously in multiple AIH gene association studies. First, *HLA-DRB1 13*:*01* and *03*:*01* alleles are known to be associated with AIH type I and *HLA-DRB1* 07:01 allele is known to be associated with AIH type II in the literature[17–20]. Molecular *HLA* typing in our samples [35] showed these *HLA-DRB1* alleles known to be associated with AIH present in our samples (Table 3). Second, a [G>GC] insertion variant within the *FTCD* gene on chromosome 21 appeared in one homozygous case in trio and one heterozygous case while this insertion is absent in the 1000G and ExAC databases and listed with MAF 0.006 in the larger gnomAD database (Table 3, S11 Table). The *FTCD* gene encodes formimidoyltransferase cyclodeaminase and is known to play a role in AIH [28]. Mutations in the *FTCD* gene on chromosome 21 have been implicated as causal for glutamate formiminotransferase deficiency, a rare metabolic disorder that affects physical and mental development[36]. The single *FTCD* [G>GC] homozygous case was inherited from her mother but missing in the father suggesting a constitutive spontaneous mutation in this type II AIH case (Table 3).

**Table 3:** *HLA-DRB1* and *FTCD* genotypes.

sample id	*HLA-DRB1 alleles*		*FTCD* [G>GC]
trio_case1	**07:01:01**	12:01:01	2/2
trio_father	10:01:01	12:01:01	1/1
trio_mother	**07:01:01**	08:01:01	1/2
case2	**13:01:01**	**13:01:01**	1/2
case3	**13:01:01**	15:01:01	1/1
case4	**13:01:01**	15:01:01	1/1
case5	11:01:02	15:01:01	1/1
case6	04:05:01	15:03:01	1/1
case7	**03:01:01**	**13:01:01**	1/1
case8	15:01:01	14:54:01	1/1

*HLA-DRB1* and FTCD G>GC insertion genotypes are shown for all samples. *HLA-DRB1* are given based on molecular typing or Illumina-X10 data when molecular typing results were not available. Alleles associated with AIH are shown in **bold**.

## Discussion

We present here the results of a comprehensive analysis of whole genome sequencing (WGS) of the St. Petersburg State University Sequencing Center at Peterhof as compared with the same samples sequenced by commercial sequencing centers outside of Russia: Illumina and Macrogen (Table 1). We compare the sequence quality, efficiency of DNA variant and genotype calling with each other and with DNA array chips of the same patients, Mendel allele transmission errors, the assessment of copy number variation and the speed of WGS throughput promised. There were slight differences in sequence coverage (Illumina was highest) and variance (Illumina was highest). For all other parameters measured (~20 in total) Peterhof and outside vendors provided very good and comparable sequence and data throughput. We must note here that while our new sequencing center produced high quality results, the time required for setting up a new sequencing facility and the cost was high as compared to the service providers outside Russia (S13 Table).

In this study we were primarily focused on WGS results comparison as the dataset used for this evaluation, consisting of eight AIH samples and two healthy parents of one case, was too small for a statistically robust association study. However, we were interested in the opportunity to use the technical replicates sequenced three times in different sequencing centers for variant prioritization. Using this replication and additional filtering steps (see Results) we identified a set of loss-of-function SNPs and short indels occurring in some of the AIH samples (S10 and S11 Tables). One of these was an insertion located in a gene *FTCD* known to be associated with autoimmune hepatitis, for which the trio case was homozygous.

Overall, the findings affirm comparable sequence data and genotyping quality in the compared centers, however differences in the timing were considerable. The success and speed of the Genome Russia Project may indeed depend on cost and speed of sequencing as expected. The AIH study lent credence to the influence of both *HLA-DRB1* and the *FTCD* association with this complex disease occurring in several patients and in different studies compared here (Table 3, S7 Table).

## Methods

### Data generation and sequencing

Ten samples from a cohort on autoimmune hepatitis were used in this work. The study was approved by the Johns Hopkins Institutional Review Board. Parents/caregivers of patients all signed informed consent prior to enrolment into the study. Blood (1–5 ml) was drawn from AIH patients and the parents of one AIH case using EDTA vacutainer tubes. Genomic DNA was isolated from 1–5 ml blood using Puregene Blood Kit chemistry on an Autopure LS automated DNA purification instrument (Qiagen, Valencia, CA) at the Johns Hopkins University Institute of Genetic Medicine Biorepository/Shipping Coordinator, GRCF Cell Center and Biorepository. Detailed description of DNA extraction protocol and chemistry are presented at https://www.qiagen.com/us/resources/resourcedetail?id=a9e6a609-4600-4b03-afbd-974318590ce5&lang=en and also https://www.qiagen.com/us/shop/sample-technologies/dna/genomic-dna/gentra-puregene-blood-kit/#productdetails. DNA concentrations were determined by spectrophotometry using a NanoDrop 1000 Spectrophotometer (Thermo Fisher Scientific, Wilmington DE).

These ten samples were sent to three sequencing centers including our local Peterhof center, Illumina (UK) and Macrogen (South Korea). Quality control during sequencing library preparation was performed by sequencing centers and was not included in sequencing center comparison.

#### Macrogen-X10

Genomic DNA libraries were prepared using TruSeq DNA PCR-Free Library Preparation Kit in accordance with TruSeq DNA PCR-free library preparation guide, producing a PCR-free library with 300–400 bp average insert size. 1 μg of each DNA sample was fragmented by Covaris system. Pairs of 151bp reads were sequenced on Illumina HiSeq X10 sequencer.

#### Illumina-X10

Illumina TruSeq PCR free sample preparation kit was used to make libraries for all 10 samples from 600ng of DNA for each sample selecting fragments with 450 bp insert size. Fragmentation was performed using Covaris system; fragments with 450 bp insert size were selected. Pairs of 151bp reads were sequenced on Illumina HiSeq X10 sequencer.

#### Illumina microarray

For validation purposes Illumina genotyped the trio using HumanOmni2.5–8 v1.2 genotyping chip.

#### Peterhof-HiSeq4000

Genomic DNA libraries were prepared using TruSeq DNA PCR-Free Library Preparation Kit (Illumina, USA). All procedures were conducted in accordance with the protocol TruSeq DNA PCR-Free Library Prep Reference Guide (2015). 1 μg of each DNA sample was used for library preparation. Clusterization of 2 nM libraries was conducted on a cBot using HiSeq 3000/4000 PE Cluster Kit (Illumina, USA). 2x150 paired-end sequencing was done by Illumina HiSeq 4000 using HiSeq 3000/4000 SBS Kit (Illumina, USA) at Peterhof, St. Petersburg State University in accordance with Illumina HiSeq 4000 System Guide (2016).

### Data quality control

Quality control (QC) was carried at the following levels:

1. Raw sequence read QC
2. QC after alignment of raw reads to human reference genome
3. Variant calling and genotyping QC

### 1. Raw read quality control

The initial quality control was performed using FastQC [37]. The distribution of 23-mer coverage was calculated and drawn by KrATER [https://pypi.python.org/pypi/KrATER/0.1] tool based on Jellyfish [38] k-mer counter. Adapter occurrence was estimated using Cookiecutter [39]. As adapter occurrence was low (less than 3% of reads, see Table 2 and S1 Table) and had little impact on the genome alignment, we skipped the adapter removal stage. Finally, only reads with mean quality equal or higher than 20 were retained. In addition, the fraction of pairs with both reads retained was estimated and examined as a QC parameter.

Five explicit parameters were measured to assess and compare the quality of sequencing data between sequencing centers (see below):

1. Estimated mean coverage (calculated only for the non-repetitive regions of genome using 23-mer distribution);
2. Variance coefficient of coverage (estimation of uniformity of coverage);
3. Fraction of read pairs with both reads retained after filtration (estimation of sequencing quality);
4. Fraction of 23-mers with errors (estimation of sequencing error rate);
5. Fraction of read pairs without adapters or "N"s (estimation of library preparation and sequencing quality).

To assess the significance of detected differences, Kruskal-Wallis and ANOVA tests (where the sequencing center was considered a fixed factor and individual sample as a random factor) were performed where applicable (for parameters No 1,2,3,4). Pairwise comparisons of estimated mean coverage (plus 95% confidence intervals) were also done between sequencing centers. All tests were performed using Stats and Lme4 R packages.

Based on raw read QC we determine whether a sample can be used in further analyses. To that aim we set the minimum or maximum value for several parameters:

1. Fraction of read pairs without adapters or Ns: F ≥ 0.95
2. Fraction of read pairs with both reads retained after filtration: F ≥ 0.95
3. Fraction of kmers with errors: F ≤ 0.15
4. Mode of coverage: C ≥ 27

If a sample fails one or more criteria, we required additional sequencing done for this sample.

### 2. Read alignment QC

We mapped raw reads that passed quality control to the GRCh38 human reference genome using bowtie2 2.2.8 [40] with the "—very-sensitive" option and obtained one BAM file per sample. Due to large insert sizes in Illumina-X10 dataset (larger than the default 500bp for bowtie2), we aligned Illumina-X10 reads with an increased insert size parameter (-X 800).

We obtained alignment statistics from BAM files using a combination of SAMtools-1.3 [41], BEDTools2-2.25.0 [42] and custom scripts written in Python 2.7. Genotype statistics was collected using BCFtools 1.3 [41]. Genotype comparison was performed using vcfeval utility from RTG Tools 3.7.1 [43].

In general we found two parameters useful for assessing differences between sequencing centers:

1. Number of reads before mapping;
2. Percentage of reads mapped.

### 3. Variant calling and genotyping QC

We sorted and indexed the individual BAM files using Sambamba 0.6.1 [44]. We used SAMtools 1.3 mpileup utility with options -q 37 -Q 30 -t AD,INFO/AD,ADF,INFO/ADF,ADR,INFO/ADR,DP,SP and BCFtools 1.3 call utility [41] with options -v -m -f GQ,GP for joint genotyping of all samples on the basis of the sorted and indexed BAM files. From the resulting VCF file we selected only SNVs that passed the following filters: (1) QUAL > 40, (2) FORMAT/GQ > 20, (3) FORMAT/DP > 10, and (4) FORMAT/SP < 20 using BCFtools view utility. We removed variants in repeated regions (as defined by RepeatMasker 4.0.5 based on Repbase Update 20140131).

We used the following main parameters to assess the quality of genotyping:

1. Number of SNVs;
2. Percentage of novel SNVs;
3. Number of indels;
4. Number of multiallelic sites;
5. Rate of Mendel inheritance errors;
6. Concordance with microarray genotypes.

#### Variant annotation and prioritization

Variant annotation was performed by Ensembl Variant Effect Predictor (VEP) release 84 [45]. Using only canonical transcripts we annotated the variants with PolyPhen [46], SIFT [47] and Condel [48] pathogenicity scores; PhyloP [49] conservation score; loss-of-function (LoF) predictions by Loss-Of-Function Transcript Effect Estimator (LOFTEE) [29]; and minor allele frequencies from 1000G [8], ExAC [34] and gnomAD [34] data. We checked the reported associations in GWAS catalog [30] and HGMD [31], performed pathogenic annotation by Gavin [32] and annotated the genes with their expression levels in liver according to GTEx [33].

To filter the LoF SNPs, we excluded variants whose genotypes failed to replicate >98% of the time in all ten individuals by three platforms (Macrogen, Illumina and Peterhof). SNPs that could not be found in databases (or had a MAF<0.01 in 1000G, ExAC, and gnomAD databases; S6 Table, S7 Table), which had >2 alternative alleles among the eight AIH cases were included. We ranked high those variants, which had a zero incidence in two healthy parents (except when the offspring case carried the allele).

In order to exclude wrong genotyping due to bowtie2 + BCFtools pipeline errors, we also ran BWA alignment followed by GATK genotyping on Illumina-X10 samples as described in GATK best practices guide [50]. We used the concordance of genotypes produced by our default pipeline as compared to the GATK pipeline to further reduce the number of false positive variants.

### CNV and SD identification

We searched for segmental duplications in the genomes sequenced with each of the three sequencing centers using the human genome assembly hg38 as reference.

The reference assembly was hard-masked from the repetitive regions using Repeat Masker and Tandem Repeat Finder software. Some other potential repeats were identified using kmer approach. The overrepresented kmers were masked out from the assembly using mapping of chromosome subregions of fixed length k = 36 onto the genome using mrFast [10] software.

The copy number (CN) values were evaluated along the chromosomes using mrCanavar [10] software in non-overlapping windows of 1Kbp of unmasked sequence. From each read of length 100 we selected two non-overlapping kmers. The flanking regions of potentially lower quality of length 9bps were excluded from the analysis.

According to the definition used in our analysis segmental duplications (SDs) are regions that span at least 10Kbp in genomic coordinates of increased average copy number value in comparison to the mean copy number value in control (non-repetitive) regions of the corresponding individual with correction for dispersion [10–12]. An SD can be considered as an aggregated segment of increased variation and as other types of polymorphism can be inherited from a common ancestor distant enough to reveal such an imprint of variation in individuals not related in three or more generations and considered unrelated in our study. The goal of SD comparison is to estimate the total level of duplications in a genome and probably identify common and unique genes and other genomic signatures affected by SDs. Segmental duplication in two or more individuals are called not overlapping if their genomic coordinates do not overlap.

### Identification of longer indels

We called genomic variants in each of Illumina-X10, Macrogen-X10 and Peterhof-HiSeq4000 samples using Platypus [51] with default options except for—assemble = 1. We filtered the obtained variants in the following series of steps: (1) indels called by Platypus (with "PASS" tag in "FILTER" field); (2) indels successfully normalized; (3) long indels (20 to 100 bp); (4) indels with quality score (QUAL) greater than 40; (5) indels with minimal genotype quality (GQ) greater than 20; (6) indels outside of low-complexity and low-mappability regions defined below. For steps (1), (2), (4), (5) we used BCFtools utilities. In step (2) we normalized indels using BCFtools norm utility with the following options:—check-ref x -m-.

In step (3) we selected long indels (20 to 100 bp) using a custom script. An indel was considered to have length of 20 to 100 bp if the difference between the lengths of the reference allele and the alternative allele was greater or equal to 20 bp and less or equal to 100 bp. In step (6) we filtered out indels located in low-complexity and low-mappability genomic regions using BEDtools intersect utility.

The regions of low mappability were identified in the following way: for each position in the genome, all 151-mers covering it were mapped back to the reference human genome using the bowtie2 aligner with the same options as used for the read alignment and the ratio of the uniquely mapped 151-mers was calculated. If the ratio was less than 0.5, then the position was considered to belong to a low-mappability region. The low-complexity genomic regions were obtained by merging three sets of regions: homopolymers of 7 bp or longer, DustMasker-identified low-complexity regions, and RepeatMasker-annotated low-complexity and microsatellite regions, and adding 10 bp to their flanks.

### HLA typing

We performed HLA genotyping using Athlates software [52] with default parameters. For that we extracted the reads mapped to HLA region and the unmapped reads and aligned them using bowtie2 [40] to the HLA database provided by Athlates. We confirmed the HLA genotypes of each individual using molecular HLA genotyping as described previously [35].

## Supporting information

S1 Fig Distribution of alternative allele counts in called genotypes. Three datasets of genotypes for 10 individuals (Illumina and Macrogen) and one dataset of genotypes for 6 individuals (Peterhof) were considered. For each variant, the number of alternative alleles was obtained; the variants were classified according to this number. Multiallelic variants were excluded from this analysis.
(PDF)

S2 Fig Distribution of copy numbers in non-duplicated (control) regions. The distributions are plotted for each sample from (A) Illumina, (B) Macrogen, (C) Peterhof.
(PDF)

S3 Fig Segmental duplications identified in trio in three datasets. "Common" bar corresponds to segmental duplications present in all three datasets.
(PDF)

S4 Fig Overlap of long indels across three sequencing centers. The Venn diagram shows the number of shared long indels in the three datasets.
(PDF)

S1 Table Comparison of various QC parameters for raw reads. Raw read quality control parameters assessed for all sequenced samples for each sequencing center.
(XLSX)

S2 Table Alignment statistics. Various parameters of alignment results are averaged over all samples in each dataset.
(DOCX)

S3 Table Statistics on called variants. Statistics on variant calling and genotyping were calculated on the 6 samples shared in the three datasets. The variants were classified as known or novel according to their presence or absence in the NCBI dbSNP database build 147.
(XLSX)

S4 Table Mendel inheritance errors. Variants violating the Mendel inheritance law were counted in the trio genotype data.
(DOCX)

S5 Table Per-sample genotype comparison between datasets. (XLSX)

S6 Table Long indel counts. The number of identified long indels is given for each sequencing center to illustrate the effect of filtering (described in the first column).
(DOCX)

S7 Table HLA genotyping and concordance of WGS-based and molecular typing. (XLSX)

S8 Table All identified LoF SNP list with annotation. (XLSX)

S9 Table All identified LoF short indel list with annotation. (XLSX)

S10 Table Filtered list of LoF SNPs. (XLSX)

S11 Table Filtered list of LoF indels. (XLSX)

S12 Table List of candidate AIH-related genes obtained from separate studies. (XLSX)

S13 Table Time estimates for 30X coverage from sequencing centers per person. (DOCX)
