# Supplementary figures and images for: Analytical “bake-off” of whole genome sequencing quality for the Genome Russia project using a small cohort for autoimmune hepatitis
Source: PLoS One. 2018 Jul 11;13(7):e0200423. doi: 10.1371/journal.pone.0200423 (PMC6040705; doi:10.1371/journal.pone.0200423)

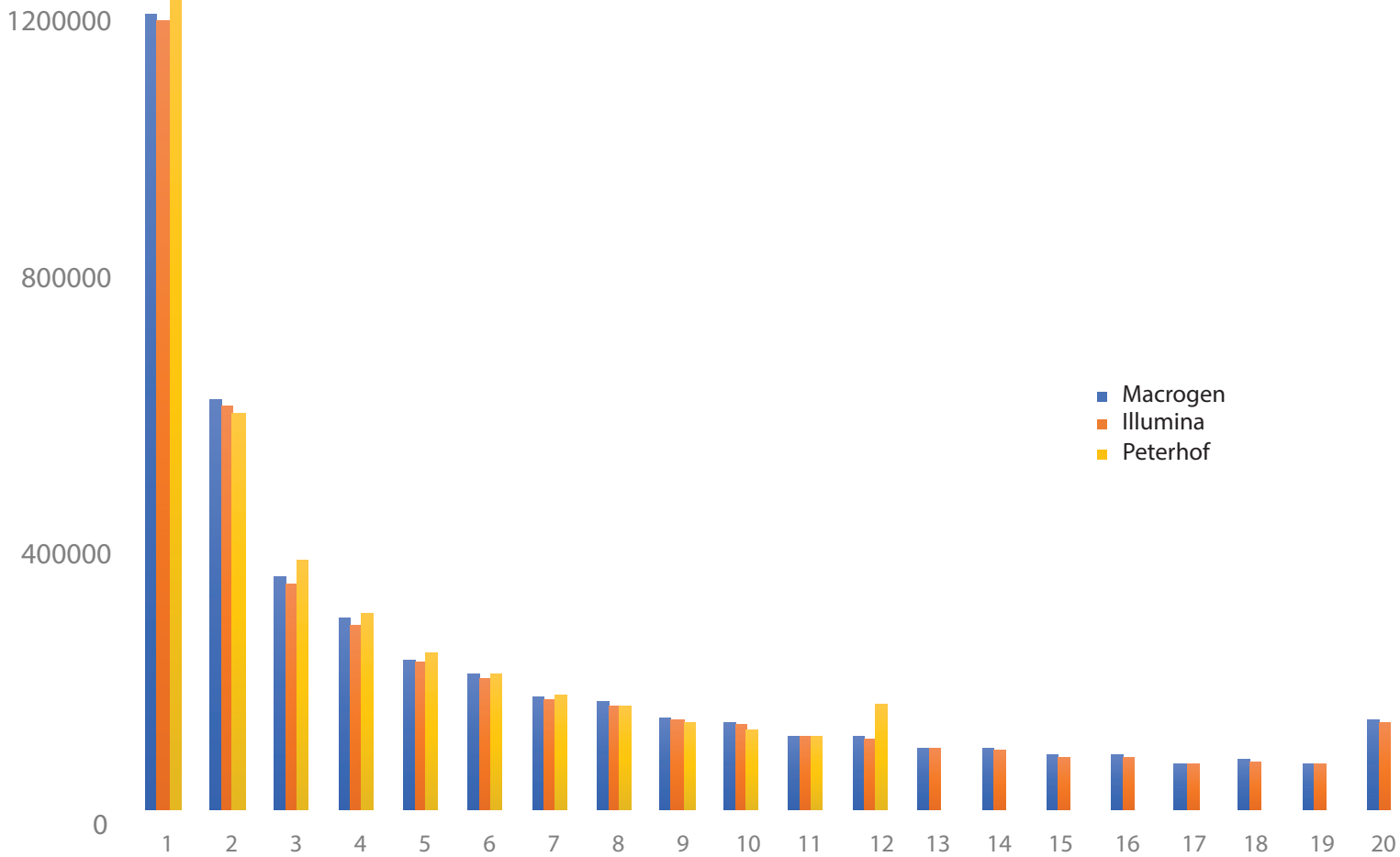

Supplement: S1 Fig — Three datasets of genotypes for 10 individuals (Illumina and Macrogen) and one dataset of genotypes for 6 individuals (Peterhof) were considered. For each variant, the number of alternative alleles was obtained; the variants were classified according to this number. Multiallelic variants were excluded from this analysis. (PDF) [file pone.0200423.s001.pdf]

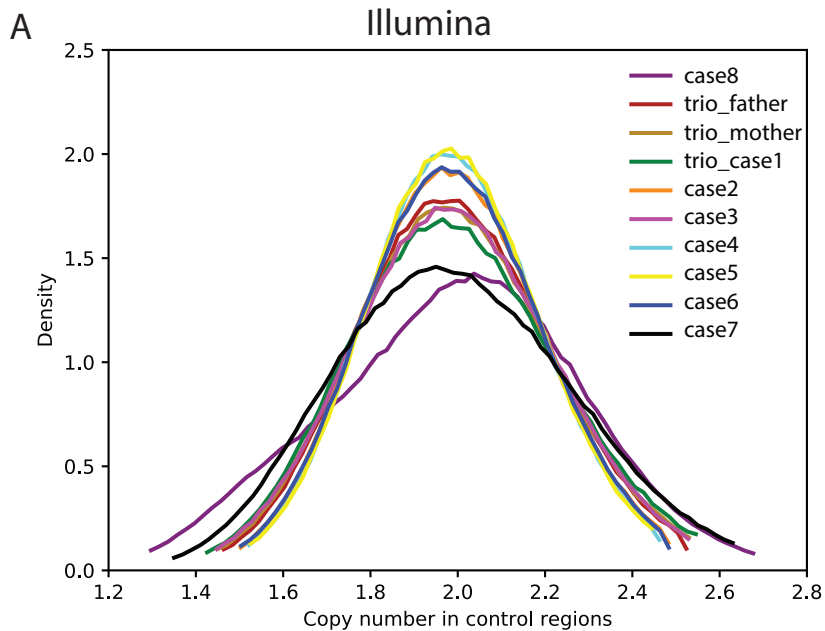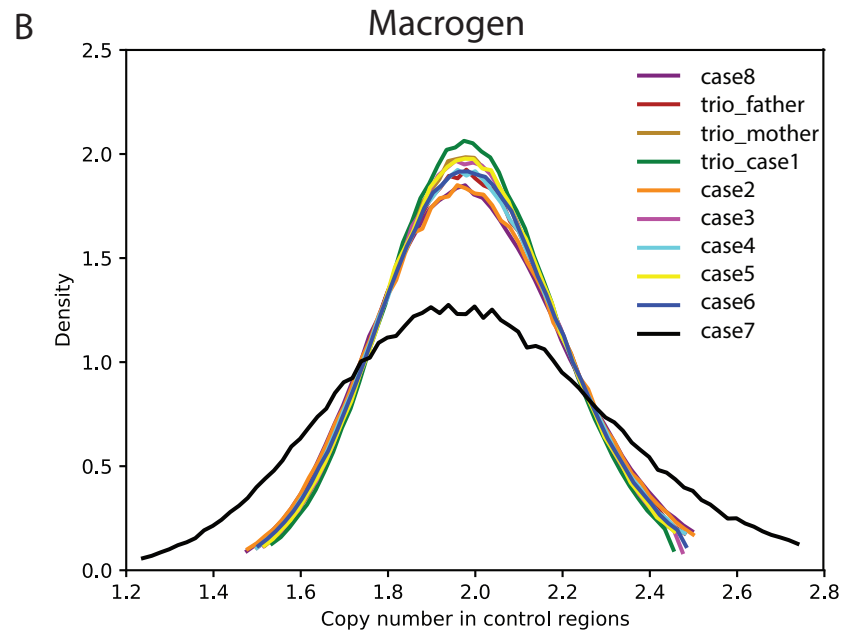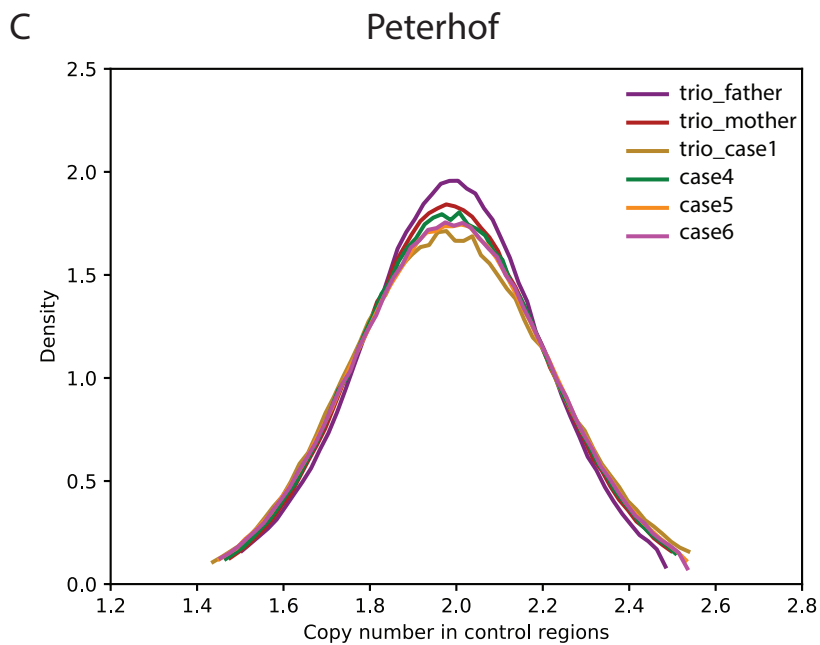

Supplement: S2 Fig — The distributions are plotted for each sample from (A) Illumina, (B) Macrogen, (C) Peterhof. (PDF) [file pone.0200423.s002.pdf]

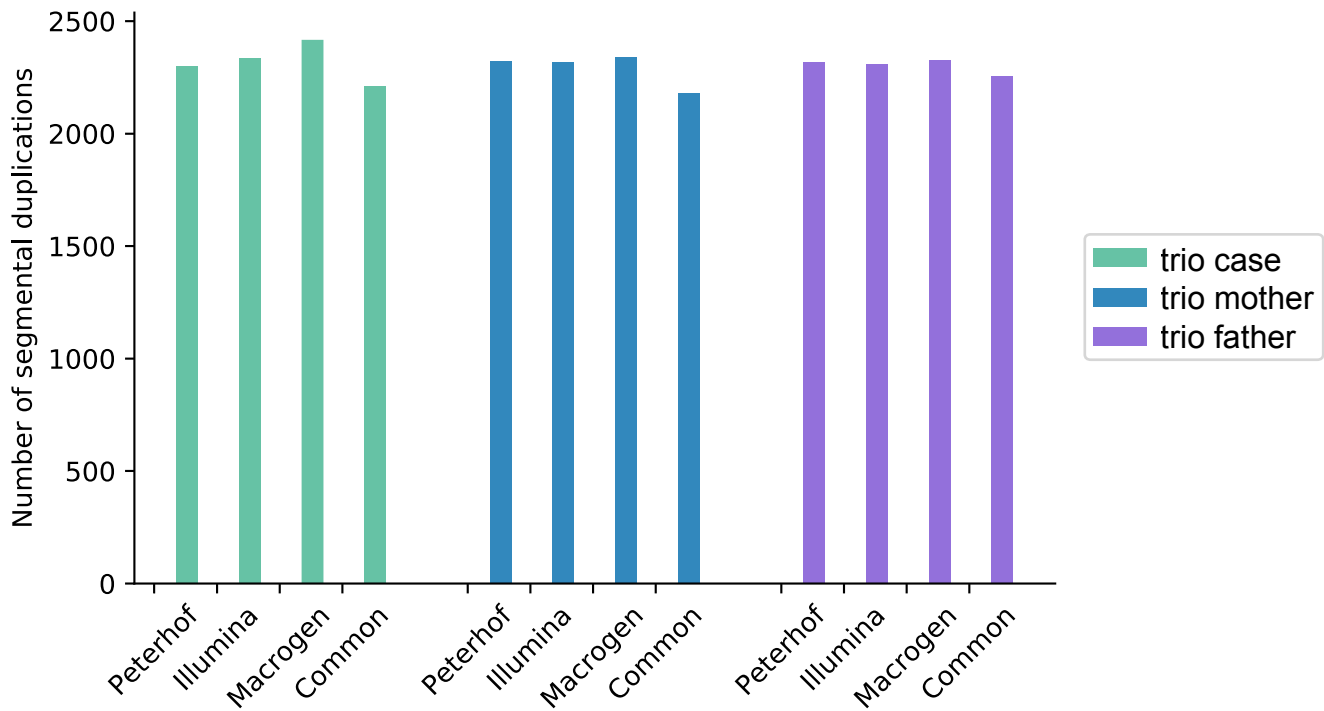

Supplement: S3 Fig — "Common" bar corresponds to segmental duplications present in all three datasets. (PDF) [file pone.0200423.s003.pdf]

Illumina

MacroGen

187

420

841

978

239

208

453

Peterhof

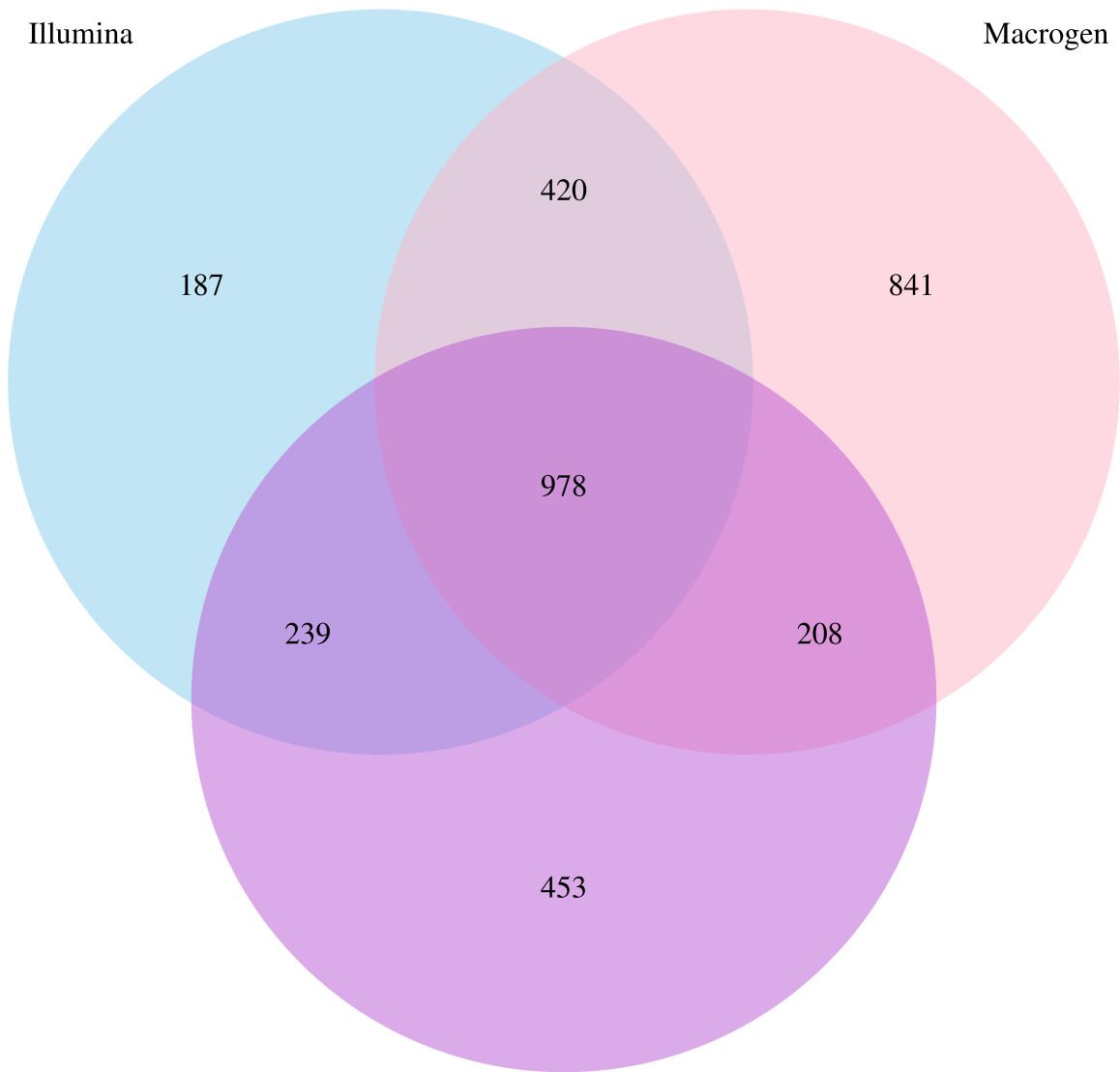

Supplement: S4 Fig — The Venn diagram shows the number of shared long indels in the three datasets. (PDF) [file pone.0200423.s004.pdf]
