# Supplementary material for: Analytical “bake-off” of whole genome sequencing quality for the Genome Russia project using a small cohort for autoimmune hepatitis
Source: PLoS One. 2018 Jul 11;13(7):e0200423. doi: 10.1371/journal.pone.0200423 (PMC6040705; doi:10.1371/journal.pone.0200423)
Supplement: S2 Table — Various parameters of alignment results are averaged over all samples in each dataset. (DOCX) [file pone.0200423.s006.docx]

**Table S2. Alignment statistics**

Various parameters of alignment results are averaged over all samples in each dataset.

The difference in the percentage of reads properly paired after alignment comes from the increased -X (insert size) parameter of bowtie2 aligner in Illumina but not in other datasets.

| **Parameter** | **Peterhof** | **Macrogen** | **Illumina** |
| --- | --- | --- | --- |
| Reads before mapping | 812,203,657 | 834,018,799 | 912,695,503 |
| Mapped reads | 794,683,499 | 810,077,813 | 889,228,650 |
| Percentage of mapped reads | 97.85 | 97.14 | 97.43 |
| Paired before alignment | 805,110,061 | 828,813,293 | 905,714,969 |
| Properly paired after alignment (%) | 82.09 | 81.31 | 95.36 |
| Reads with itself and mate mapped | 780,328,927 | 798,415,154 | 873,973,266 |
| Singletons | 7,705,249 | 6,797,079 | 8,571,697 |
| Singletons (%) | 0.96 | 0.82 | 0.95 |
| Reads with mate mapped to a different chromosome | 4,087,727 | 4,659,902 | 4,590,735 |
| Genome coverage | 41.64 | 42.86 | 58.91 |
| Median insert size | 404.67 | 398.4 | 440.50 |
| Min insert size | 30.33 | 125.8 | 124.80 |
| Max insert size | 240,465,486 | 241,570,002 | 240,628,069 |
| Mean insert size | 407.84 | 403.54 | 444.40 |
| Insert size standard deviation | 85.5 | 88.47 | 105.34 |
