# Supplementary material for: Analytical “bake-off” of whole genome sequencing quality for the Genome Russia project using a small cohort for autoimmune hepatitis
Source: PLoS One. 2018 Jul 11;13(7):e0200423. doi: 10.1371/journal.pone.0200423 (PMC6040705; doi:10.1371/journal.pone.0200423)
Supplement: S4 Table — Variants violating the Mendel inheritance law were counted in the trio genotype data. (DOCX) [file pone.0200423.s008.docx]

**Table S4. Mendel inheritance errors**

Variants violating the Mendel inheritance law were counted in the trio genotype data.

|  | **# Consistent** | **# Inconsistent** | **% Mendel errors** |
| --- | --- | --- | --- |
| **Illumina** | 4,526,706 | 26,288 | 0.58% |
| **Macrogen** | 4,330,894 | 13,012 | 0.30% |
| **Peterhof** | 3,848,600 | 10,491 | 0.27% |
| **Illumina-chip** | 2,246,849 | 176 | 0.01% |
