# Supplementary material for: Analytical “bake-off” of whole genome sequencing quality for the Genome Russia project using a small cohort for autoimmune hepatitis
Source: PLoS One. 2018 Jul 11;13(7):e0200423. doi: 10.1371/journal.pone.0200423 (PMC6040705; doi:10.1371/journal.pone.0200423)
Supplement: S6 Table — The number of identified long indels is given for each sequencing center to illustrate the effect of filtering (described in the first column). (DOCX) [file pone.0200423.s010.docx]

**Table S6. Long indel counts**

The number of identified long indels is given for each sequencing center to illustrate the effect of filtering (described in the first column).

|  | **Illumina-X10** | **Macrogen-X10** | **Peterhof-HiSeq4000** |
| --- | --- | --- | --- |
| Number of samples | 10 | 10 | 6 |
| Indels, normalized | 865,294 | 909,257 | 656,008 |
| Indels, normalized, 20-100 bp | 4,923 | 4,977 | 3,687 |
| Indels, normalized, 20-100 bp, QUAL>40 | 4,922 | 4,976 | 3,687 |
| Indels, normalized, 20-100 bp, QUAL>40, GQ>20 | 4,415 | 4,448 | 3,470 |
| Indels, normalized, 20-100 bp, QUAL>40, GQ>20, mappability | 2,508 | 2,447 | 1,878 |
