# Supplementary material for: Analytical “bake-off” of whole genome sequencing quality for the Genome Russia project using a small cohort for autoimmune hepatitis
Source: PLoS One. 2018 Jul 11;13(7):e0200423. doi: 10.1371/journal.pone.0200423 (PMC6040705; doi:10.1371/journal.pone.0200423)
Supplement: S13 Table — (DOCX) [file pone.0200423.s017.docx]

**Table S13. Time estimates for 30X coverage from sequencing centers per person**

| Contact | Center | Platform | Location | Coverage | Estimated time for 100WGS |
| --- | --- | --- | --- | --- | --- |
| Nick Bates | Illumina | Illumina HiSeq X10 | Essex UK | 30X | NR |
| Woon-Ah Seo | Macrogen | Illumina HiSeq X10 | Seoul Korea | 30X | 30 days |
| Andrey Glotov | Peterhof | Illumina HiSeq 4000 | Peterhof, Russia | 30X | 70 days |

NR - not reported
